# Supplementary material for: Structural insights into dsRNA processing by Drosophila Dicer-2–Loqs-PD
Source: Nature. 2022 Jun 29;607(7918):399–406. doi: 10.1038/s41586-022-04911-x (PMC9279154; doi:10.1038/s41586-022-04911-x)
Supplement: Supplementary file 1 — Uncropped gels. The red boxes represent the area displayed in figures. a, SDS–PAGE gels of the pull-down result for Dcr-2 with WT and three variants of His-tagged Loqs-PD. The red box shows the area for Fig. 2h–i, and the yellow box shows the area for Fig. 2i. b, SDS–PAGE gel of the size-exclusion chromatography result of Dcr-2–Loqs-PD–50 bp dsRNA complex for Extended Data Fig. 1c. c, 12% polyacrylamide denaturing gel for Extended Data Fig. 1d. d, 16% polyacrylamide denaturing gel for Extended Data Fig. 9a. e, 16% polyacrylamide denaturing gel for Extended Data Fig. 10a. f, 8% native gel for Extended Data Fig. 10b. g, 16% polyacrylamide denaturing gel for Extended Data Fig. 10c. [file 41586_2022_4911_MOESM1_ESM.pdf]

nature portfolio

https://doi.org/10.1038/s41586-022-04911-x

Supplementary information

Structural insights into dsRNA processing by *Drosophila* Dicer-2–Loqs-PD

In the format provided by the authors and unedited

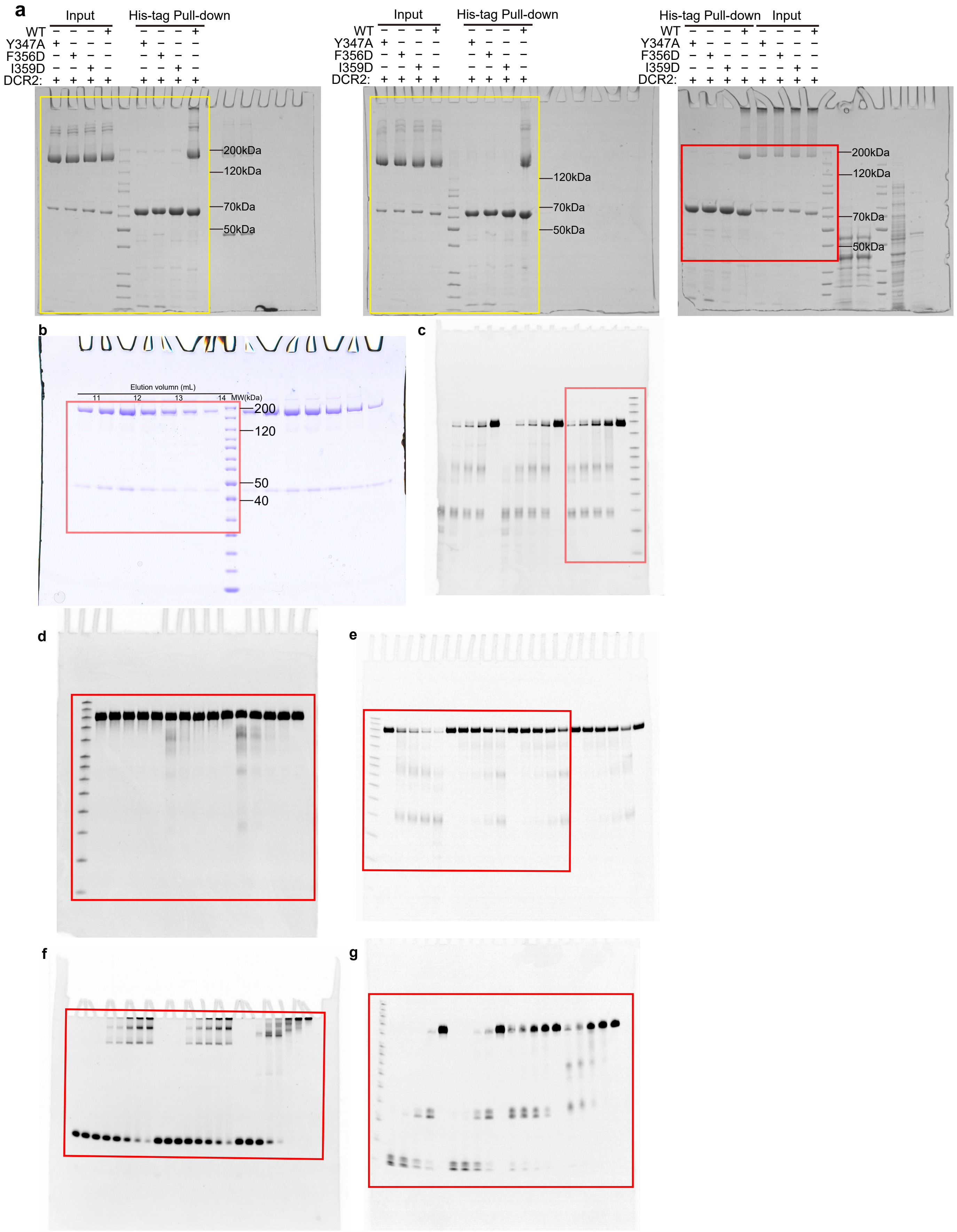
a
Input
His-tag Pull-down
WT − − − + − − − +
Y347A + − − − + − − −
F356D − + − − − + − −
I359D − − + − − − + −
DCR2: + + + + + + + +
200kDa
120kDa
70kDa
50kDa
Input
His-tag Pull-down
WT − − − + − − − +
Y347A + − − − + − − −
F356D − + − − − + − −
I359D − − + − − − + −
DCR2: + + + + + + + +
120kDa
70kDa
50kDa
His-tag Pull-down
Input
WT − − − + − − − +
Y347A + − − − + − − −
F356D − + − − − + − −
I359D − − + − − − + −
DCR2: + + + + + + + +
200kDa
120kDa
70kDa
50kDa
b
Elution volumn (mL)
11
12
13
14
MW(kDa)
200
120
50
40
c
d
e
f
g
