# Supplementary material for: Structural insights into dsRNA processing by Drosophila Dicer-2–Loqs-PD
Source: Nature. 2022 Jun 29;607(7918):399–406. doi: 10.1038/s41586-022-04911-x (PMC9279154; doi:10.1038/s41586-022-04911-x)
Supplement: Supplementary file 2 — Reporting Summary [file 41586_2022_4911_MOESM2_ESM.pdf]

nature portfolio

Corresponding author(s): Hong-Wei Wang

Last updated by author(s): Apr 20, 2022

Reporting Summary

Nature Portfolio wishes to improve the reproducibility of the work that we publish. This form provides structure for consistency and transparency in reporting. For further information on Nature Portfolio policies, see our Editorial Policies and the Editorial Policy Checklist.

Statistics

For all statistical analyses, confirm that the following items are present in the figure legend, table legend, main text, or Methods section.

n/a	Confirmed	
☐	☒	The exact sample size (n) for each experimental group/condition, given as a discrete number and unit of measurement
☒	☐	A statement on whether measurements were taken from distinct samples or whether the same sample was measured repeatedly
☐	☒	The statistical test(s) used AND whether they are one- or two-sided<br>*Only common tests should be described solely by name; describe more complex techniques in the Methods section.*
☒	☐	A description of all covariates tested
☒	☐	A description of any assumptions or corrections, such as tests of normality and adjustment for multiple comparisons
☐	☒	A full description of the statistical parameters including central tendency (e.g. means) or other basic estimates (e.g. regression coefficient) AND variation (e.g. standard deviation) or associated estimates of uncertainty (e.g. confidence intervals)
☐	☒	For null hypothesis testing, the test statistic (e.g. F, t, r) with confidence intervals, effect sizes, degrees of freedom and P value noted<br>*Give P values as exact values whenever suitable.*
☒	☐	For Bayesian analysis, information on the choice of priors and Markov chain Monte Carlo settings
☒	☐	For hierarchical and complex designs, identification of the appropriate level for tests and full reporting of outcomes
☒	☐	Estimates of effect sizes (e.g. Cohen's d, Pearson's r), indicating how they were calculated

Our web collection on statistics for biologists contains articles on many of the points above.

Software and code

Policy information about availability of computer code

Data collection	We used AutoEMation (version 2.0) to collect cryo-EM datasets of Dcr-2/LoqsPD and dsRNA in apo, initial binding, translocation and dicing states, written by Dr. Jianlin Lei at Tsinghua University. Post-dicing state dataset was collected by EPU 2 of Thermo Fisher Scientific.
Data analysis	We used MotionCor2 (version 1.1.0) to correct the beam-induced motion of cryo-EM micrographs. The CTF values of these motion-corrected micrographs were determined by CTFFIND4 algorithm (version 4.15). We used Relion (version 3.1.3) and Cryo-SPARC (version 3.20) to perform image analysis and 3D reconstruction. Local resolution distribution was evaluated using Bsoft(version 2.07) software package. We used COOT (version 0.9.4) for de novo model building and adjusted the model with COOT and ISOLDE (version 1.1). All the models were refined against the EM map by PHENIX(version 1.19.2). The structural analysis was performed in UCSF Chimera (version 1.13.1) and ChimeraX (version 1.2.5). All these softwares are open-source except Cryo-SPARC, but it is free for educational users.<br>Cross-linked peptides were identified and evaluated using pLink2(version 2.3.9) software.

For manuscripts utilizing custom algorithms or software that are central to the research but not yet described in published literature, software must be made available to editors and reviewers. We strongly encourage code deposition in a community repository (e.g. GitHub). See the Nature Portfolio guidelines for submitting code & software for further information.

Data

Policy information about availability of data

All manuscripts must include a data availability statement. This statement should provide the following information, where applicable:

- Accession codes, unique identifiers, or web links for publicly available datasets
- A description of any restrictions on data availability
- For clinical datasets or third party data, please ensure that the statement adheres to our policy

The atomic coordinates and structure factors in apo and initial dsRNA binding states, early- and mid-translocation states, active-dicing and post-dicing states in this study have been deposited in the RCSB Protein Data Bank (PDB) and Electron Microscopy Data Bank (EMDB) under EMD accession codes 32236, 32237, 32238, 32239, 32240, 32241 and PDB ID codes 7W0A, 7W0B, 7W0C, 7W0D, 7W0E, 7W0F, respectively. The PDB and EMDB codes are also listed in Extended Data Table 1. For uncropped gel images, see Supplementary Figure 1. Other structures used in this study were retrieved from PDB with accession code 7VG2 (AtDCL3), 2EZ6 (AaRNase III), 2FFL (GiDicer), 5ZAK (HsDicer), 6LXD (HsDrosha), 5F9H (HsRIG-I), 7ELE (AtDCL1). The information of Dcr-2 and Loqs-PD could be found in Uniprot database with code A1ZAW0 and M9MRT5. Any other data or materials can be obtained from the corresponding author upon reasonable request.

Field-specific reporting

Please select the one below that is the best fit for your research. If you are not sure, read the appropriate sections before making your selection.

☒ Life sciences ☐ Behavioural & social sciences ☐ Ecological, evolutionary & environmental sciences

For a reference copy of the document with all sections, see nature.com/documents/nr-reporting-summary-flat.pdf

Life sciences study design

All studies must disclose on these points even when the disclosure is negative.

Sample size	No sample size calculation was performed. For the cryo-EM maps, we ended up collecting data when we thought the structures were good enough or cannot be improved any more. For biochemical assays, we performed two to three replicates since the replications are successful.
Data exclusions	For cryo-EM reconstruction, particles grouped in bad classes with poorly defined features were excluded, because these particles were normally denatured or dissociated samples, which were harmful for high-resolution 3D reconstruction.
Replication	Our native gel shift assays, pull-down assays, and activity assays were performed in two to three independent replicates, all attempts at replication were successful. No data was excluded.
Randomization	Samples were allocated random, including the particle-motion and structural determination.
Blinding	For cryo-EM reconstruction, particles were randomly divided into two parts, and used for 3D structure determination. The consistence of structures generated by these two sub-datasets was used for the blinding test. For experiments other than cryo-EM, allowcation of samples into experiment groups was not performed in this study, therefore blinding was not relevant to our biochemical experiments.

Reporting for specific materials, systems and methods

We require information from authors about some types of materials, experimental systems and methods used in many studies. Here, indicate whether each material, system or method listed is relevant to your study. If you are not sure if a list item applies to your research, read the appropriate section before selecting a response.

Materials & experimental systems

n/a	Involved in the study	
☒	☐	Antibodies
☐	☒	Eukaryotic cell lines
☒	☐	Palaeontology and archaeology
☒	☐	Animals and other organisms
☒	☐	Human research participants
☒	☐	Clinical data
☒	☐	Dual use research of concern

Methods

n/a	Involved in the study	
☒	☐	ChIP-seq
☒	☐	Flow cytometry
☒	☐	MRI-based neuroimaging

Eukaryotic cell lines

Policy information about cell lines

Cell line source(s)	SF9, obtained from Invitrogen.

Authentication	The cell lines were obtained from commercial source and none of lines used were authenticated.
Mycoplasma contamination	Cell lines in this study were negative to mycoplasma by detection by PCR.
Commonly misidentified lines (See ICLAC register)	No commonly misidentified cell lines were used in this study.
